# Characterization of circulating miRNAs in the treatment of primary liver tumors

**DOI:** 10.1002/cnr2.1964

**Published:** 2023-12-25

**Authors:** Tomohiro Umezu, Shogo Tanaka, Shoji Kubo, Masaru Enomoto, Akihiro Tamori, Takahiro Ochiya, Y.‐H. Taguchi, Masahiko Kuroda, Yoshiki Murakami

**Affiliations:** ^1^ Department of Molecular Pathology Tokyo Medical University Tokyo Japan; ^2^ Department of Hepato‐Biliary‐Pancreatic Surgery Osaka Metropolitan University, Graduate School of Medicine Osaka Japan; ^3^ Department of Hepatology, Graduate School of Medicine Osaka Metropolitan University, Graduate School of Medicine Osaka Japan; ^4^ Department of Molecular and Cellular Medicine, Institute of Medical Science Tokyo Medical University Tokyo Japan; ^5^ Department of Physics Chuo University Tokyo Japan; ^6^ Department of Dentistry Asahi University Gifu Japan

**Keywords:** biliary tract cancer, direct acting antiviral treatment, extracellular vesicle, hepatocellular carcinoma, miRNA, sustained viral response

## Abstract

**Background and Aim:**

Circulating micro RNAs (miRNAs) indicate clinical pathologies such as inflammation and carcinogenesis. In this study, we aimed to investigate whether miRNA expression level patterns in could be used to diagnose hepatocellular carcinoma (HCC) and biliary tract cancer (BTC), and the relationship miRNA expression patterns and cancer etiology.

**Methods:**

Patients with HCC and BTC with indications for surgery were selected for the study. Total RNA was extracted from the extracellular vesicle (EV)‐rich fraction of the serum and analyzed using Toray miRNA microarray. Samples were divided into two cohorts in order of collection, the first 85 HCC were analyzed using a microarray based on miRBase ver.2.0 (hereafter v20 cohort), and the second 177 HCC and 43 BTC were analyzed using a microarray based on miRBase ver.21 (hereafter v21 cohort).

**Results:**

Using miRNA expression patterns, we found that HCC and BTC could be identified with an area under curve (AUC) 0.754 (v21 cohort). Patients with anti‐hepatitis C virus (HCV) treatment (SVR‐HCC) and without antiviral treatment (HCV‐HCC) could be distinguished by an AUC 0.811 (v20 cohort) and AUC 0.798 (v21 cohort), respectively.

**Conclusions:**

In this study, we could diagnose primary hepatic malignant tumor using miRNA expression patterns. Moreover, the difference of miRNA expression in SVR‐HCC and HCV‐HCC can be important information for enclosing cases that are prone to carcinogenesis after being cured with antiviral agents, but also for uncovering the mechanism for some carcinogenic potential remains even after persistent virus infection has disappeared.

## 1 INTRODUCTION

Hepatocellular carcinoma (HCC) and biliary tract cancer (BTC) are typical primary malignant tumors of the liver. They have different origins and are generally distinguished by tumor markers and diagnostic imaging. However, there are also similarities between the two: Hepatitis C Virus (HCV) and Hepatitis B Virus (HBV) infection is a risk factor not only for HCC, but also for BTC.^1^ Among different forms of HCC, there is a pathologically mixed HCC in which cholangiocarcinoma (CCC) tissue is also present, known as combined cancer.^2^ It is difficult to distinguish between the two using tumor markers or diagnostic images from computed tomography and magnetic resonance imaging scans if not typical cases. Although no miRNA‐based differentiation between HCC and BTC has been reported to date, changes in circulating N‐linked glycosylation are associated with various cancers such as gastric cancer^3^ and lung cancer.^4^ Recent attempts have been made to differentiate between HCC and BTC using six types of N‐glycans.^5^

As far as HCC is concerned, the recent availability of direct‐acting antiviral agents (DAAs) has enabled viral clearance in almost all patients with HCV infection.^6^, ^7^, ^8^ The annual incidence of HCC in patients with chronic hepatitis is less than 1% with patients who had a sustained virologic response (SVR).^9^ However, the annual rate of hepatocarcinogenesis in patients with cirrhosis is 3%–7%.^10^, ^11^, ^12^ Even if SVR is acquired in this way, the risk of carcinogenesis varies depending on the degree of the primary disease, and the risk of carcinogenesis is not low. There is a need for continuous follow‐up for carcinogenesis even after acquiring SVR.

Acquisition of SVR using antiviral drugs means that the viral infection is cured, and it also means that the virus does not reactivate. However, since the carcinogenic potential has not completely disappeared as described above, the characteristics of carcinogenic cases from cases that have acquired SVR and methods for predicting carcinogenesis are being actively developed. For example, machine learning was used create a prediction algorithm to predict cancer recurrence with an accuracy of 88.5% by analyzing the extracellular vesicle (hereafter EV) rich fraction miRNA expression pattern of patients with liver cirrhosis who acquired SVR using DAA as HCC treatment. In our previous works, the cancer prediction rate for HCC‐naïve patients with chronic liver disease is 85.5%.^13^ Furthermore, when hepatocellular carcinogenesis prediction was performed using age, Alanine Aminotransferase (ALT) value, and alpha fetoprotein (αFP) value, the cases with subjects 65 years or younger, and ALT<30, αFP <5 are predicted to not develop cancer after 5 years of follow‐up.^14^ Efforts are being made to predict HCC at an early stage and also assess the degree of liver fibrosis and inflammation^15^, ^16^, ^17^, ^18^ using circulating miRNA analysis. New biomarkers have been reported, such as post‐treatment prediction methods.

Using information miRNA expression from EV fractions, we aimed to analyze a method for distinguishing between HCC and BTC, elucidation of the characteristics of carcinogenesis in HCC, and a method for predicting postoperative recurrence of HCC and BTC.

## 2 MATERIALS AND METHODS

### 2.1 Sample information

Serum samples before surgical treatment (pre) and after surgical treatment (post1 and post2) were obtained from 85, 74 to 47 patients with HCC, respectively. Post1 and post 2 samples refer to the serum samples taken after operation within 14 days, and beyond 14 days, respectively (Table 1). These samples were analyzed using 3D‐Gene Human miRNA Oligo Chip based on miRbase ver. 20 (Toray Industries, Inc., Kanagawa, Japan). Serum before surgical treatment was also obtained from 177 patients with HCC and 43 patients with BTC (41 CCC and 2 gaLL bladder cancer). These samples were analyzed using 3D‐Gene Human miRNA Oligo Chip based on miRbase ver. 21.

**TABLE 1:** Summary of clinical feature.

Cohort	Group	Sex	Age	Stage	Recurrence
v20	**HCC pre**	M:58, F:27	68.53 ± 9.45	I:42, II:24, III:15, IV:3, ND:1	<1:25, 1 < <2: 12, <2: 48
v20	**HCC post1**	M:54, F20	68.56 ± 8.77	I:39, II:18, III:13, IV:3, ND:1	<1:20, 1 < <2:7, 2<:47
v20	**HCC post2**	M:31, F:16	67.83 ± 10.31	I:22, II:10, III:11, IV:3, ND:1	<1:15, 1 < <2: 5, 2<:27
v21	**HCC pre**	M:131, F:46	69.64 ± 12.22	I:76, II:63, III:22, IV:12, ND:4	<1:40, 1 < <2: 42, <2: 86, ND: 9
v21	**BTC**	M:26, F:16, ND:1	63.83 ± 13.83	I:3, II:5, III:14, IV:19, ND:2	1<:4, 1 < <2: 4, 2<:26, ND:9

Group	Etiology	AFP (ng/mL)	DCP (mAU/mL)	CA19‐9 (U/mL)	CEA (ng/mL)
HCC pre	ALD:6, HBV:21, HCV:45, NBNC:13	2690.32 ± 14730.86	8133.20 ± 58340.62	15.82 ± 16.44	3.51 ± 3.30
HCC post1	ALD:4, HBV:19, HCV:39, NBNC:12	1640.28 ± 10079.15	9258.07 ± 62495.87	14.93 ± 15.73	3.68 ± 3.09
HCC post2	ALD:3, HBV:11, HCV:25, NBNC:8	4739.62 ± 19659.22	14340.85 ± 78270.99	16.07 ± 16.58	2.81 ± 1.90
HCC pre	ALD:5, HBV:37, HBV + HCV:3, HCV:90, NBNC:42	3050.18 ± 31898.52	3317.48 ± 11449.91	25.32 ± 101.40	4.07 ± 6.80
BTC	ALD:1, HBV:2, HCV:2, ND:38	8.14 ± 8.64	33.12 ± 27.50	1359.05 ± 6632.78	4.11 ± 4.61

Abbreviations: 1 < <2, recurrence between 1 and 2 years after surgical treatment; 2<, no recurrence within 2 years; ALD, alcoholic liver disease; HBV + HCV, HBV and HCV coinfection; NBNC, neither HBV nor HCV infection; ND, not determined; NI, no information; Recurrence, <1, within 1 year recurrence after surgical treatment.

### 2.2 RNA preparation and microarray analysis

RNA from the EV‐rich fraction was prepared using ExoQuick (System Biosciences, Palo Alto, CA). RNA was extracted using a miRNeasy Mini Kit (Qiagen, Hilden, Germany). Sixty nanograms of total RNA were analyzed using the 3D‐Gene miRNA microarray RNA extraction reagent from the liquid sample kit (Toray Industries, Inc., Kanagawa, Japan). Comprehensive miRNA expression analysis was performed by using two types of 3D‐Gene miRNA Labeling Kit and 3D‐Gene Human miRNA Oligo Chip (Toray Industries, Inc.). They correspond to miRbase release 20 (2555 miRNA published) and miRbase release 21 (2588 miRNA published). Since the microarray was upgraded from miRbase release 20 to 21 during the analysis plan, the cohort v20 (hereafter v20 cohort) was the group of which was analyzed with the miRbase release 20 microarray, and the cohort v21 (hereafter v21 cohort) was the group of which was analyzed with the miRbase release 21 microarray. All microarray data for this study conformed to the “Minimum Information about a Microarray Experiment guidelines” and are publicly available in the GEO database GSE212211.

### 2.3 Statistical analysis

Tensor Decomposition Based Unsupervised Feature Extraction (TD based UFE) was used to extract unsupervised features of each group using the respective miRNA expression information obtained by microarray.

Suppose that *x*_*ij*_ represents the expression of *i*th microRNA of *j*th sample. *x*_*ij*_ is normalized as.

$$\sum_{i=1}^{N}x_{\mathrm{ij}}=0$$

$$\sum_{i=1}^{N}x_{\mathrm{ij}}^{2}=N$$

We apply principal component analysis to xij and we got principal component score *u*_*li*_

$$\sum_{i^{\prime }=1}^{N}\left(\sum_{j=1}^{M}x_{\mathrm{ij}}x_{i\prime j}\right)u_{\mathrm{li}\prime }=\lambda_{l}u_{\mathrm{li}}$$

and principal component loading *v*_*lj*_ as

$$v_{\mathrm{lj}}=\sum_{i=1}^{N}x_{\mathrm{ij}}u_{\mathrm{li}}$$

In order to identify which js are associated with the distinction between two classes, we applied regression analysis to vlj as

$$v_{\mathrm{lj}}=a_{l}+\sum_{s=1}^{2}b_{\mathrm{ls}}\delta_{\mathrm{js}}$$

where al and bls are regression coefficients and δjs taken when j belongs to *s* th class otherwise 0. *P*‐values are corrected with BH criterion. ls associated with adjusted *P*‐values less than .05 are selected. Then we found that l ∈ Ωl are associated with adjusted *P*‐values less than .05.

After identifying vlj, which is distinct between two classes, we attribute *P*‐values to *i* as

$$P_{i}=P_{\chi^{2}}\left[>\sum_{l=1}^{2}{\left(\frac{u_{\mathrm{li}}}{\sigma_{l}}\right)}^{2}\right]$$

where P_(χ^2) [>x] is the cumulative χ2 distribution where argument is larger than x. *P*‐values are corrected by BH criterion and is associated with adjusted *P*‐values less than .01 are selected.

After selecting ith, we recomputed vlj using the selected is. Using recomputed vlj (l ∈ Ωl), js are discriminated with liner discriminant analysis.

For Q2, Ωl = 5. For Q8, at first, Ωl = 2 is used. Ωl = 2,4 is used after re‐computation of vlj. For Q6, at first, Ωl = 10 is used. Ωl = 11,13 is used after re‐computation of vlj.

## 3 RESULTS

### 3.1 Diagnostic analysis of cancer by miRNA

**FIGURE 1:**
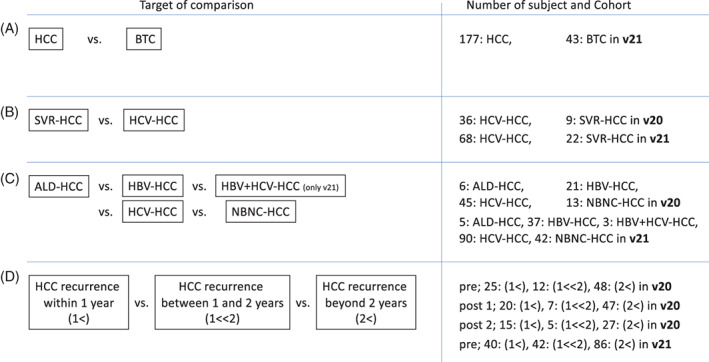
Analysis design of this study. (A) Comparison of miRNA expression in EV between HCC and BTC. (B) Comparison of miRNA expression pattern in EV between HCV‐HCC and SVR‐HCC. (C) Comparison of miRNA expression pattern in EV among ALD‐HCC, HBV‐HCC, HCV‐HCC, NBNC‐HCC, and HBV + HCV‐HCC. HBV + HCV‐HCC analyzed only in the v21 cohort. (D) Comparison of miRNA expression pattern in EV among recurrence period of HCC resection. In v20 cohort, analysis was performed using before operation (pre), after operation within 14 days (post 1) and after operation beyond 14 days (post 2) specimens, and in v21, analysis was performed using pre specimens.

**FIGURE 2:**
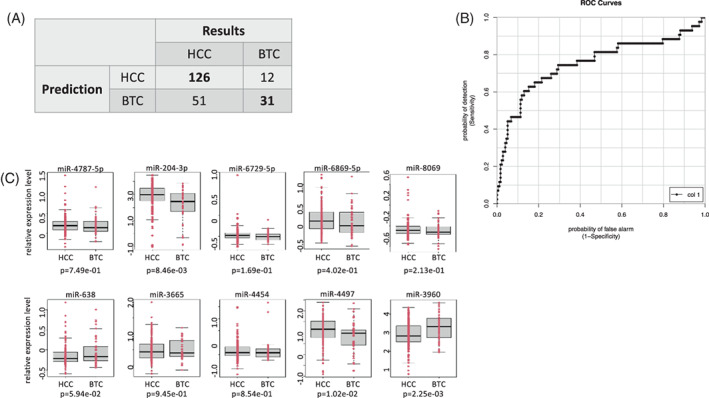
Discrimination between HCC and BTC. (A) Discrimination between HCC and BTC using cohort v21 was performed using information of 10 miRNA expression patterns. 177 cases were diagnosed as HCC based on the miRNA expression pattern, of which 126 were HCC and 51 were BTC. In addition, 43 patients were diagnosed with BTC, 12 with HCC, and 31 with BTC. The number of cases in which predictions and results matched are shown in bold. (B) This discriminant of ROC curve is shown. (C) Expression levels of miRNAs used for discrimination in each group, *p*‐value was also shown. Red dots indicate individual miRNA expression levels.

In the v21 cohort, analyzed using microarrays conforming to mirbase ver. 21, we attempted to differentiate between HCC and BTC using samples from the pre‐group using miRNA expression pattern by Tensor Decomposition Based Unsupervised Feature Extraction (TD based UFE). Based on the expression patterns of 10 miRNAs (hsa‐miR‐638, 92a‐2‐5p, 762, 1260b, 3178, 3196, 3663‐3p, 3665, 4454, 4463), 177 cases were predicted to be HCC, of which 126 were correctly identified; 43 were predicted to be BTC, of which 31 were correctly identified. The AUC for this analysis was 0.754 (Figures 1A, 2 and Table 1). Among these 10 miRNAs, there were 3 miRNAs (miR‐204‐3p, 4497, and 3960) with *p* < .05 or less between both groups, and other 7 miRNAs had no significant difference. The miRNAs selected here were not those with large differences between the two groups, but rather these 10 miRNAs were obtained in order to obtain the maximum discrimination ability between the two groups.

### 3.2 Characteristics of miRNAs by carcinogenesis etiology

**FIGURE 3:**
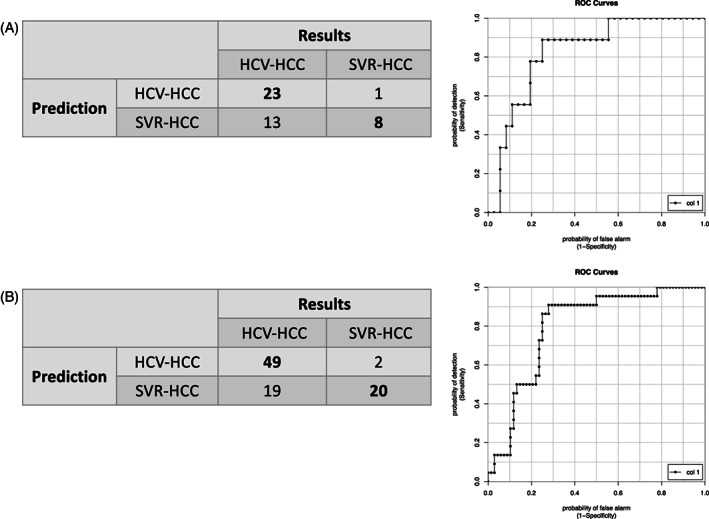
Discrimination between HCV‐HCC and SVR‐HCC. (A) Using the 19 miRNAs shown in supplementary Figure 1, we distinguished HCV‐HCC and SVR‐HCC from cases used in cohort v20. The left table shows the prediction result. Using the expression information of 19 miRNAs, 24 cases were predicted to have the cause of carcinogenesis as HCV‐HCC, 23 cases were HCV‐HCC as expected, and one case was SVR‐HCC contrary to expectations. Next, there were 21 cases in which the cause of carcinogenesis was predicted to be SVR‐HCC, 8 cases in which the cause of carcinogenesis was SVR‐HCC as expected, and 13 cases in which contrary to predictions the cause was HCV‐HCC. Right panel shows AUC curve of this prediction. The number of cases in which predictions and results matched are shown in bold. (B) Using the 19 miRNAs shown in supplementary Figure 2, we distinguished HCV‐HCC and SVR‐HCC from cases used in cohort v21. The left table shows the prediction result. Using the expression information of 19 miRNAs, 51 cases were predicted to have the cause of carcinogenesis as HCV‐HCC, 49 cases were HCV‐HCC as expected, and 2 cases was SVR‐HCC contrary to expectations. Next, there were 39 cases in which the cause of carcinogenesis was predicted to be SVR‐HCC, 20 cases in which the cause of carcinogenesis was SVR‐HCC as expected, and 19 cases in which contrary to predictions the cause was HCV‐HCC. Right panel shows AUC curve of this prediction.

First, using an analysis of sera collected from 45 patients with HCC (v20 cohort analyzed using microarrays conforming to mirbase ver. 20) before resection, miRNA expression patterns were compared between sera obtained from 36 patients with HCV‐caused HCC (hereafter HCV‐HCC) and 9 patients with HCC in HCV‐treated cases (hereafter SVR‐HCC). Expression patterns of 19 miRNAs (hsa‐miR‐3178, 4294, 3648, 3665, 4488, 4497, 4516, 3960, 4745‐5p, 4787‐5p, 204‐3p, 1233‐5p, 5787, 6089, 6727‐5p, 6786‐5p, 6869‐5p, 7704, and 8069) were used to distinguish between HCV‐HCC and SVR‐HCC. Twenty‐four HCV‐HCC cases were predicted from their miRNA expression patterns, and 23 cases were correct. Similarly, 21 cases of SVR‐HCC were predicted from miRNA expression patterns, and 8 were correct. The AUC for this analysis was 0.811 (Figures 1B and 3A, Supplementary Figure 1, and Table 1). Looking at the 19 miRNAs used to differentiate between HCV‐HCC and SVR‐HCC individually, only miR‐3665 showed *p* < .05, and the other 18 miRNAs have not achieved *p* < .05. Although it is not possible to distinguish between the two groups using information on individual miRNAs, for example miR‐4488, when information on 19 miRNAs is combined using Tensor decomposition, the fine discrimination results are obtained. In univariate analysis of the two groups of HCV‐HCC and SVR‐HCC, the difference of expression in several miRNAs are shown *p* < .05 or less, however, the discriminative ability of this analysis is significantly lower than that of analysis using Tensor decomposition.

There are 68 HCV‐HCC and 22 SVR‐HCC cases in the v21 cohort, HCV‐HCC and SVR‐HCC were differentiated by using 19 miRNAs expression pattern (hsa‐miR‐451a, 638, 3648, 3665, 4488, 4497, 4516, 3960, 4745‐5p, 4787‐5p, 204‐3p, 1233‐5p, 5787, 6089, 6090, 6131, 6727‐5p, 6869‐5p, and 8072). The miRNA expression patterns identified 51 HCV‐HCC cases, where 49 cases were correctly predicted. Furthermore, 39 cases were predicted to be SVR‐HCC where 20 cases were correctly predicted (Figures 1B, 3B, Supplementary Figure 2). Twelve miRNAs (hsa‐miR‐3648, 3665, 4488, 4497, 4516, 3960, 4745‐5p, 4787‐5p, 204‐3p, 1233‐5p, 5787, 6089, and 6727‐5p) were used for discrimination between HCV‐HCC and SVR‐HCC not only v20 but also v21 cohort. When using the v21 cohort, when looking at the 19 miRNAs used to discriminate between HCV‐HCC and SVR‐HCC individually, those showing *p* < .05 were miR‐3648, 4497, 4516, and 4745‐5p., 204‐3p, 1233‐5p, 5787, and 6869‐5p, and the other 11 miRNAs did not achieve *p* < .05. With same theory, these 19 combinations are necessary to obtain good discrimination.

Eighty‐five HCCs from the v20 cohort were used to characterize miRNA expression by cause of carcinogenesis. The breakdown was 6 alcoholic liver disease (ALD)‐related, 21 HBV‐related, 45 HCV‐related, and 13 neither HBV nor HCV infection (NBNC). No miRNA expression pattern was found that characterized these four groups (Figure 1B). Using 222 HCC samples from the v21 cohort, we investigated the characteristics of miRNA expression by cause of carcinogenesis. The breakdown was 5 ALD, 37 HBV‐related, 90 HCV‐related, 3 HBV + HCV co‐infection, and 42 NBNC. No miRNA expression patterns characterized by carcinogenesis were found. (Figure 1C).

### 3.3 Recurrence prediction after cancer resection by miRNA expression pattern

Finally, we examined whether the v20 cohort could be used to predict recurrence time after resection of the HCC tumor. Eighty‐five HCC samples collected before surgery (pre) were used. Twenty‐five had recurrence within 1 year (<1), 12 had recurrence for more than 1 year and less than 2 years (1<, <2), and 48 had no recurrence for more than 2 years (<2). We then analyzed 71 HCC cases collected within 14 days after surgery (post 1). Twenty had recurrence within 1 year, 7 had recurrence over 1 year and less than 2 years, and 44 had no recurrence over 2 years. Furthermore, we analyzed 50 HCC cases collected 14 days or more after surgery (post 2). Fifteen had recurrence within 1 year, 5 had recurrence within 1 to 2 years, and 30 had no recurrence within 2 years or more. Using the v21 cohort, we analyzed 168 HCC cases collected before surgery (pre). 40 had recurrence within 1 year, 42 had recurrence over 1 year and less than 2 years, and 86 had no recurrence over 2 years (Figure 1D). Using any cohorts, miRNA expression patterns could not predict recurrence prediction time.

## 4 DISCUSSION

We analyzed the miRNA expression pattern in the blood serum of patients with HCC and BTC for primary liver tumors that are indicated for surgery, before and after surgery. We found that it was possible to distinguish between HCC and BTC cases using miRNA collected before surgery. We believe that it is important to accurately differentiate between BTC and HCC in a non‐invasive manner. Typical cases can be easily differentiated based on images and histopathology, but there are cases in which it is difficult to differentiate even when histopathology is also evaluated. Differentiating between BTC and HCC may be clinically difficult. The infection of hepatitis virus (HBV and HCV) has been well known not risk factor only in HCC but also in BTC.^19^ Moreover, there are also cases in which there is a histopathological concept of a mixed carcinoma of both BTC and HCC.^20^ In addition, there are some cases where sampling is difficult under echo guidance because they exist near structures such as blood vessels and bile ducts. Given this situation, we believe that deriving a method to differentiate BTC and HCC using blood information would be clinically useful.

But to date, there has been no report on the differentiation between HCC and BTC using blood miRNA expression patterns. Therefore, this study is novel in reporting the differentiation between HCC and BTC using miRNA expression patterns. There are several reports on the prognosis and diagnosis of HCC using 8 miRNAs (miR‐320b, 6724‐5p, 6877‐5p, 4448, 4749‐5p, 663a, 4651, and 6885‐5p) in the blood that can be used to diagnose HCC at its early stage.^18^ On the other hands, in diagnosing ICC, the combination of low blood miR‐150 level and CA19‐9 level improved the diagnostic ability.^21^ Low expression of serum miR‐1281, miR‐126, miR‐26a, miR‐30b and miR‐122 compared to healthy subjects.^22^ miR‐122 is associated with postoperative prognosis in ICC.^23^ Until now, studies have only compared cancer with normal or benign diseases, whether HCC or BTC, and direct comparison between HCC and BTC has not been performed. Therefore, we reviewed the results of this analysis based on the previous comparison of HCC and BTC in tumor tissues.^24^ Comprehensive transcriptome analysis and metabolomic analysis were reported for classifying four groups of not only HCC and BTC, but also corresponding non‐cancerous areas. 17 miRNA expression patterns (let‐7a‐5p, let‐7b‐5p, miR‐16‐5p, 21‐5p, 29a‐3p, 122‐5p, 451a, 642a‐3p, 3917, 3960, 4286, 4454, 4459, 4516, 5100, 6087, and 6089) were used for classifying the four groups.^24^ miR‐4454 is a common miRNA used in tissue and blood studies that are useful for differentiation of HCC and BTC. In particular, increased circulating miR‐4454 expression positively correlated with prognosis post hepatocellular carcinoma treatment.^25^ MiR‐4454 promotes carcinogenesis in liver tissues,^26^ and its mode of involvement in carcinogenesis differs between blood and tissue.

With the development of HCV treatment, many patients with chronic liver disease with hepatitis C can achieve SVR. However, even if virus‐related proteins are not continuously supply, hepatocarcinogenesis is observed with a certain probability. In this analysis, the expression patterns of miRNA in EV were clearly different between HCV‐HCC and SVR‐HCC samples. Analysis of miRNAs expression in cancer tissues with SVR‐HCC and HCV‐HCC, and whose expression are elevated in HCV carcinogenesis compared with SVR carcinogenesis include miR‐130a, 30a‐3p, 100, 134, 139‐5p, 144, 150, 192, and 451a). Previous our reports showed that miR‐130a,^27^ and 134^28^ are “so‐called” oncomiRs involved in carcinogenesis promotion, when overexpressed. However, miR‐30a‐3p,^29^ 100,^30^ 139‐5p,^31^ 144,^32^ 150,^33^ 451a,^34^ 192^35^ have been reported as anti‐oncomiRs that suppress carcinogenesis when overexpressed. The down‐regulated miRNAs in HCV carcinogenesis compared with SVR carcinogenesis include miR‐18a, 19a, 21, 30d, 93, 146a, 181, 494, 24, 26a, 26b, 27a, 92a, 127, 142‐3p, and 145a.^36^ Since both HCV‐HCC and SVR‐HCC represent cancer cases, it is inferred that the miRNA expression patterns are different between HCV‐treated and un‐treated cases. The miRNA expression patterns in the HCV‐HCC and SVR‐HCC tumor tissues as previously reported^36^ were not related to the circulating miRNA pattern analyzed in this study. Furthermore, there were no specific features in miRNA expression patterns based on the antiviral treatments used in achieving to SVR (Table 2). Recurrence is a problem in HCC treatment. There is an important concept of eliminating HCV, which is the cause of HCC, and both AASLD and EASL have treatment guidelines for direct treatment with viral agents if HCV remains after HCC treatment. It has also been reported that PDL‐1 treatment of HCC differs between viral HCC and NASH‐derived HCC.^37^ These findings suggest that the HCV infection status may influence the selection of anticancer treatment and the recurrence of HCC. Based on the above, the differentiation between HCV‐HCC and SVR‐HCC is useful information for analyzing responsiveness to cancer treatment and recurrence mode, and is considered useful for analyzing carcinogenic mechanisms.

**TABLE 2:** Regimen of SVR acquisition treatment.

Treatment	Case No.	Treatment	Case No.
IFN	8	PEG‐IFN + RBV + TLV	2
IFNα	2	ASV + DCV	1
IFNα+RBV	4	SOF + LDV	5
PEG‐IFN	1	SOV + RBV	4
PEG‐IFN + RBV	4		

Abbreviations: ASV, asunaprevir; DCV, daclatasvir; IFN, interferon alpha; IFN, interferon; LDV, ledipasvir; PEG‐IFN, pegylated interferon; RBV, ribavirin; SOF, sofosbuvir; TLV, telaprevir.

We investigated the relationship between the condition before cancer and after cancer. For prediction of HCC recurrence in patients with liver cirrhosis who had a history of cancer and who developed SVR using antiviral agents, the expression patterns of four miRNAs (hsa‐miR‐4718, 6511a‐5p, 642a‐5, and 4448) were used. Four miRNAs (hsa‐miR‐211‐3p, 6826‐3p, 1236‐3p, 4448) were used to predict HCC recurrence in chronic hepatitis (CH) and liver cirrhosis (LC) patients with a history of cancer who developed SVR using antiviral drugs. Four miRNAs (hsa‐miR‐762, 8069, 7847‐3p, 7846‐493p) were used for predict HCC occurrence in CH and LC patients with no history of cancer and who developed SVR after using antiviral drugs.^13^ miR‐6090 was used to predict carcinogenesis and to differentiate between HCV‐HCC and SVR‐HCC. The expression of miR‐6090 is enhanced by ionizing irradiation^38^ and is associated with stroke in cardiovascular disease.^39^ This suggests that abnormal expression of miR‐6090 is induced as a result of acute or chronic inflammation.

In this study, Taguchi newly developed TD based UFE to perform unsupervised feature extraction. This method is applicable to gene expression, DNA methylation, and histone modification etc. It can perform multi‐omics analysis. EV‐rich fractions were collected before and after surgery for primary hepatic malignant tumors, and miRNA oncogenesis analysis was performed. HCV‐HCC and SVR‐HCC had different miRNA expression patterns. It is also potentially applicable to single cell omics data sets. We have already reported a method to predict SVR carcinogenesis as well as to identify miRNAs related to SVR carcinogenesis by performing miRNA expression analysis of EV collected before carcinogenesis in SVR cases.^13^ Together, these data reveal that miRNAs present in EV differ not only before and after carcinogenesis, but also according to the mode of carcinogenesis. We further identified miRNAs that distinguish between HCC and BTC. Regarding the treatment of HCC, it has been shown that the reactivity of the drugs used differs depending on the cause of carcinogenesis.^37^ The fact that peripheral blood miRNA profiles differ depending on the cause of carcinogenesis is expected to be useful in elucidating the mechanisms of carcinogenesis and therapeutic response. Analysis of miRNA expression in EV can be useful for follow‐up observation of chronic liver disease in anticipation of carcinogenesis prevention. Finally, when comparing two groups, some differences in individual gene expression are not significant, and one of the limitations of this analysis is that the function of individual genes cannot be compared between groups. However, considering that pathway analysis is currently being used as a mainstream method, our analysis method uses multiple gene expression rather than individual gene expression, so it can be said to be appropriate.

## AUTHOR CONTRIBUTIONS

Tomohiro Umezu, Shogo Tanaka and Yoshiki Murakami performed the experiments, intellectual input and discussions. Shoji Kubo, Masaru Enomoto and Akihiro Tamori helped write the manuscript, and provided discussions. Takahiro Ochiya, Y.‐H. Taguchi and Masahiko Kuroda supervised the research and provided helpful discussion. Y.‐H. Taguchi provided chemometric support and analyzed data. Yoshiki Murakami designed the experiments, analyzed and interpreted data, and wrote the manuscript.

## FUNDING INFORMATION

This work was supported by Program on the Innovative Development and the Application of New Drugs for Hepatitis B (JP22fk0310503).

## CONFLICT OF INTEREST STATEMENT

The authors have no competing interests to declare.

## ETHICS STATEMENT

This study was conducted according to the guidelines of the 1975 Declaration of Helsinki (2013 version).

Approval of the research protocol and Registry and the Registration No. of the study/trial: The study protocol was approved by the Ethics Committees of Osaka City University Hospital (Project code No. 1358, approved on June 12, 2008).

## INFORMED CONSENT

Written informed consent was obtained from all patients prior to treatment.

## Supporting information


**Data S1:** Supporting Information.

## Data Availability

Data sharing is not applicable to this article as no new data were created or analyzed in this study.

## References

[1] Wang Y , Yuan Y , Gu D . Hepatitis B and C virus infections and the risk of biliary tract cancers: a meta‐analysis of observational studies. Infect Agent Cancer. 2022;17:45. 36030232 10.1186/s13027-022-00457-9 PMC9420284

[2] Rimassa L , Personeni N , Czauderna C , Foerster F , Galle P . Systemic treatment of HCC in special populations. J Hepatol. 2021;74:931‐943. 33248171 10.1016/j.jhep.2020.11.026

[3] Kodar K , Stadlmann J , Klaamas K , Sergeyev B , Kurtenkov O . Immunoglobulin G fc N‐glycan profiling in patients with gastric cancer by LC‐ESI‐MS: relation to tumor progression and survival. Glycoconj J. 2012;29:57‐66. 22179780 10.1007/s10719-011-9364-z

[4] Chen G , Wang Y , Qin X , et al. Change in IgG1 fc N‐linked glycosylation in human lung cancer: age‐ and sex‐related diagnostic potential. Electrophoresis. 2013;34:2407‐2416. 23766031 10.1002/elps.201200455

[5] Huang C , Xu X , Wang M , et al. Serum N‐glycan fingerprint helps to discriminate intrahepatic cholangiocarcinoma from hepatocellular carcinoma. Electrophoresis. 2021;42:1187‐1195. 33570803 10.1002/elps.202000392

[6] Afdhal N , Reddy KR , Nelson DR , et al. Ledipasvir and sofosbuvir for previously treated HCV genotype 1 infection. N Engl J Med. 2014;370:1483‐1493. 24725238 10.1056/NEJMoa1316366

[7] Feld JJ , Jacobson IM , Hezode C , et al. Sofosbuvir and Velpatasvir for HCV genotype 1, 2, 4, 5, and 6 infection. N Engl J Med. 2015;373:2599‐2607. 26571066 10.1056/NEJMoa1512610

[8] Tahata Y , Hikita H , Mochida S , et al. Sofosbuvir plus velpatasvir treatment for hepatitis C virus in patients with decompensated cirrhosis: a Japanese real‐world multicenter study. J Gastroenterol. 2021;56:67‐77. 33001338 10.1007/s00535-020-01733-4

[9] Kanwal F , Kramer JR , Asch SM , Cao Y , Li L , El‐Serag HB . Long‐term risk of hepatocellular carcinoma in HCV patients treated with direct acting antiviral agents. Hepatology. 2020;71:44‐55. 31222774 10.1002/hep.30823

[10] Ogawa E , Furusyo N , Nomura H , et al. Short‐term risk of hepatocellular carcinoma after hepatitis C virus eradication following direct‐acting anti‐viral treatment. Aliment Pharmacol Ther. 2018;47:104‐113. 29035002 10.1111/apt.14380

[11] Ide T , Koga H , Nakano M , et al. Direct‐acting antiviral agents do not increase the incidence of hepatocellular carcinoma development: a prospective, multicenter study. Hepatol Int. 2019;13:293‐301. 30820753 10.1007/s12072-019-09939-2

[12] Abe K , Wakabayashi H , Nakayama H , et al. Factors associated with hepatocellular carcinoma occurrence after HCV eradication in patients without cirrhosis or with compensated cirrhosis. PLoS One. 2020;15:e0243473. 33284844 10.1371/journal.pone.0243473 PMC7721183

[13] Itami‐Matsumoto S , Hayakawa M , Uchida‐Kobayashi S , et al. Circulating Exosomal miRNA profiles predict the occurrence and recurrence of hepatocellular carcinoma in patients with direct‐acting antiviral‐induced sustained viral response. Biomedicine. 2019;7:7. 10.3390/biomedicines7040087 PMC6966514 31684167

[14] Tahata Y , Sakamori R , Yamada R , et al. Risk of hepatocellular carcinoma after sustained virologic response in hepatitis C virus patients without advanced liver fibrosis. Hepatol Res. 2022;52:824‐832. 35749289 10.1111/hepr.13806

[15] Murakami Y , Toyoda H , Tanahashi T , et al. Comprehensive miRNA expression analysis in peripheral blood can diagnose liver disease. PLoS One. 2012;7:e48366. 23152743 10.1371/journal.pone.0048366 PMC3485241

[16] Calvente CJ , Tameda M , Johnson CD , et al. Neutrophils contribute to spontaneous resolution of liver inflammation and fibrosis via microRNA‐223. J Clin Invest. 2019;129:4091‐4109. 31295147 10.1172/JCI122258 PMC6763256

[17] Umezu T , Tsuneyama K , Kanekura K , et al. Comprehensive analysis of liver and blood miRNA in precancerous conditions. Sci Rep. 2020;10:21766. 33303811 10.1038/s41598-020-78500-1 PMC7728755

[18] Yamamoto Y , Kondo S , Matsuzaki J , et al. Highly sensitive circulating MicroRNA panel for accurate detection of hepatocellular carcinoma in patients with liver disease. Hepatol Commun. 2020;4:284‐297. 32025611 10.1002/hep4.1451 PMC6996324

[19] Kaneko S , Kurosaki M , Kurisu A , Akita T , Tanaka J , Kanto T . Impact of antiviral therapy for disease progression and non‐invasive liver fibrosis index in patients with chronic hepatitis C: Markov chain model analysis. Hepatol Res. 2022;52:665‐676. 35591759 10.1111/hepr.13794

[20] Razumilava N , Gores GJ . Cholangiocarcinoma. Lancet. 2014;383:2168‐2179. 24581682 10.1016/S0140-6736(13)61903-0 PMC4069226

[21] Salem PES , Ghazala RA , El Gendi AM , Emara DM , Ahmed NM . The association between circulating MicroRNA‐150 level and cholangiocarcinoma. J Clin Lab Anal. 2020;34:e23397. 33161598 10.1002/jcla.23397 PMC7676191

[22] Voigtlander T , Gupta SK , Thum S , et al. MicroRNAs in serum and bile of patients with primary sclerosing cholangitis and/or cholangiocarcinoma. PLoS One. 2015;10:e0139305. 26431155 10.1371/journal.pone.0139305 PMC4591993

[23] Loosen SH , Lurje G , Wiltberger G , et al. Serum levels of miR‐29, miR‐122, miR‐155 and miR‐192 are elevated in patients with cholangiocarcinoma. PLoS One. 2019;14:e0210944. 30653586 10.1371/journal.pone.0210944 PMC6336320

[24] Murakami Y , Kubo S , Tamori A , et al. Comprehensive analysis of transcriptome and metabolome analysis in intrahepatic cholangiocarcinoma and hepatocellular carcinoma. Sci Rep. 2015;5:16294. 26538415 10.1038/srep16294 PMC4633735

[25] Pratama MY , Visintin A , Croce LS , Tiribelli C , Pascut D . Circulatory miRNA as a biomarker for therapy response and disease‐free survival in hepatocellular carcinoma. Cancers. 2020;12:12. 10.3390/cancers12102810 PMC7601056 33003646

[26] Lin H , Zhang R , Wu W , Lei L . miR‐4454 promotes hepatic carcinoma progression by targeting Vps4A and Rab27A. Oxid Med Cell Longev. 2021;24:9230435. 10.1155/2021/9230435 PMC8580624 34777698

[27] Zhou Y , Li R , Yu H , Wang R , Shen Z . microRNA‐130a is an oncomir suppressing the expression of CRMP4 in gastric cancer. Onco Targets Ther. 2017;10:3893‐3905. 28831264 10.2147/OTT.S139443 PMC5548272

[28] Peng SY , Tu HF , Yang CC , et al. miR‐134 targets PDCD7 to reduce E‐cadherin expression and enhance oral cancer progression. Int J Cancer. 2018;143:2892‐2904. 29971778 10.1002/ijc.31638

[29] Wei D , Yu G , Zhao Y . MicroRNA‐30a‐3p inhibits the progression of lung cancer via the PI3K/AKT by targeting DNA methyltransferase 3a. Onco Targets Ther. 2019;12:7015‐7024. 31695416 10.2147/OTT.S213583 PMC6717841

[30] Chen D , Sun Y , Yuan Y , et al. miR‐100 induces epithelial‐mesenchymal transition but suppresses tumorigenesis, migration and invasion. PLoS Genet. 2014;10:e1004177. 24586203 10.1371/journal.pgen.1004177 PMC3937226

[31] Zhao Y , Tao Q , Li S , Zheng P , Liu J , Liang X . Both endogenous and exogenous miR‐139‐5p inhibit fusobacterium nucleatum‐related colorectal cancer development. Eur J Pharmacol. 2020;888:173459. 32768506 10.1016/j.ejphar.2020.173459

[32] Shao Y , Li P , Zhu ST , et al. MiR‐26a and miR‐144 inhibit proliferation and metastasis of esophageal squamous cell cancer by inhibiting cyclooxygenase‐2. Oncotarget. 2016;7:15173‐15186. 26959737 10.18632/oncotarget.7908 PMC4924778

[33] Li Z , Zhou X , Huang J , et al. MicroRNA hsa‐miR‐150‐5p inhibits nasopharyngeal carcinogenesis by suppressing PYCR1 (pyrroline‐5‐carboxylate reductase 1). Bioengineered. 2021;12:9766‐9778. 34696668 10.1080/21655979.2021.1995102 PMC8810012

[34] Zhang H , Chen P , Yang J . miR‐451a suppresses the development of breast cancer via targeted inhibition of CCND2. Mol Cell Probes. 2020;54:101651. 32828867 10.1016/j.mcp.2020.101651

[35] Gu Y , Wei X , Sun Y , et al. miR‐192‐5p silencing by genetic aberrations is a key event in hepatocellular carcinomas with cancer stem cell features. Cancer Res. 2019;79:941‐953. 30530815 10.1158/0008-5472.CAN-18-1675 PMC6397664

[36] Tamori A , Murakami Y , Kubo S , et al. MicroRNA expression in hepatocellular carcinoma after the eradication of chronic hepatitis virus C infection using interferon therapy. Hepatol Res. 2016;46:E26‐E35. 25788219 10.1111/hepr.12518

[37] Pfister D , Nunez NG , Pinyol R , et al. NASH limits anti‐tumour surveillance in immunotherapy‐treated HCC. Nature. 2021;592:450‐456. 33762733 10.1038/s41586-021-03362-0 PMC8046670

[38] Song M , Xie D , Gao S , et al. A biomarker panel of radiation‐upregulated miRNA as signature for ionizing radiation exposure. Life. 2020;10:10. 33352926 10.3390/life10120361 PMC7766228

[39] Sonoda T , Matsuzaki J , Yamamoto Y , et al. Serum MicroRNA‐based risk prediction for stroke. Stroke. 2019;50:1510‐1518. 31136284 10.1161/STROKEAHA.118.023648

