# Supplementary material for: Characterization of circulating miRNAs in the treatment of primary liver tumors
Source: Cancer Rep (Hoboken). 2023 Dec 25;7(2):e1964. doi: 10.1002/cnr2.1964 (PMC10849994; doi:10.1002/cnr2.1964)
Supplement: Supplementary file 1 — Data S1: Supporting Information. [file CNR2-7-e1964-s001.pdf]

List of Supporting Information:

Supplementary table 1. Detail clinical information

Supplementary figure 1. Expression level of 19 miRNAs used discrimination between HCV-HCC and SVR-HCC (v20 cohort).

miRNA expression pattern and p-value were also shown and Red dots indicate individual miRNA expression levels.

Supplementary figure 2. Expression level of 19 miRNAs used discrimination between HCV-HCC and SVR-HCC (v21 cohort)

miRNA expression pattern and p-value were also shown and Red dots indicate individual miRNA expression levels.

Supplementary table 1. Detail clinical information in this study

				pefore treatment					microarray	recurrence	etiology	HCV treatment	sampling time
Description	Code No.	age	sex	AFP	DCP	CA19-9	CEA	stage					
HCC	HCC002pre	81	F	42.9	609	5	2.8	I	v20	<1	HCV	no treatment	before operation
HCC	HCC003pre	76	F	18.6	41	10	1.4	III	v20	2<	HBV		before operation
HCC	HCC006pre	69	F	862.9	909	ND	ND	I	v20	<1	NBNC		before operation
HCC	HCC018pre	62	M	989.5	93762	ND	ND	ND	v20	<1	HCV	no treatment	before operation
HCC	HCC019pre	65	M	8.1	40	ND	4.7	I	v20	2<	HBV		before operation
HCC	HCC024pre	76	M	2.3	12	6	1.3	I	v20	2<	HCV	SVR	before operation
HCC	HCC026pre	70	F	10.8	18	ND	ND	I	v20	2<	HCV	no treatment	before operation
HCC	HCC028pre	72	M	3.6	112	ND	ND	I	v20	2<	HCV	no treatment	before operation
HCC	HCC032pre	73	F	30.2	10	21	3.2	I	v20	2<	HCV	no treatment	before operation
HCC	HCC034pre	60	M	103.8	1220	ND	ND	III	v20	1<<2	NBNC		before operation
HCC	HCC037pre	80	M	4.5	191	4	7.2	III	v20	<1	HCV	no treatment	before operation
HCC	HCC038pre	79	M	52.2	1000	5	1	II	v20	2<	HCV	no treatment	before operation
HCC	HCC040pre	65	M	1113.3	3148	18	1.5	IV	v20	2<	HBV		before operation
HCC	HCC041pre	76	M	4	333	9	ND	II	v20	1<<2	ALD		before operation
HCC	HCC043pre	60	F	1.8	16	ND	ND	I	v20	2<	HCV	no treatment	before operation
HCC	HCC044pre	45	F	1264.4	4831	ND	ND	III	v20	2<	HBV		before operation
HCC	HCC046pre	63	M	3.7	21	9	2.8	II	v20	2<	HBV		before operation
HCC	HCC049pre	49	M	7.2	46	ND	ND	I	v20	<1	ALD		before operation
HCC	HCC050pre	63	F	3.5	27	10	0.6	II	v20	2<	NBNC		before operation
HCC	HCC051pre	76	M	3.1	12	4	2	II	v20	2<	NBNC		before operation
HCC	HCC052pre	75	M	4	5190	16	1.6	III	v20	<1	HCV	no treatment	before operation
HCC	HCC053pre	51	M	3.9	19	12	1.4	II	v20	<1	HBV		before operation
HCC	HCC054pre	69	M	52.7	58	7	1.4	I	v20	2<	HBV		before operation
HCC	HCC058pre	70	M	3	34	ND	ND	I	v20	1<<2	HBV		before operation
HCC	HCC060pre	76	F	1078.4	847	ND	ND	II	v20	1<<2	HCV	SVR	before operation
HCC	HCC061pre	77	F	11.7	265	24	5.9	II	v20	<1	HBV		before operation
HCC	HCC066pre	63	M	217.2	403	ND	ND	III	v20	<1	HCV	no treatment	before operation
HCC	HCC070pre	67	M	20	59	6	1.4	I	v20	2<	HBV		before operation
HCC	HCC071pre	73	M	4.1	38	5.6	ND	II	v20	1<<2	HCV	no treatment	before operation
HCC	HCC072pre	65	M	10.1	28	ND	ND	I	v20	2<	HCV	SVR	before operation
HCC	HCC074pre	67	M	2.5	37	61	3.9	II	v20	2<	HCV	SVR	before operation
HCC	HCC075pre	77	F	12	14	ND	ND	I	v20	1<<2	HCV	no treatment	before operation
HCC	HCC076pre	76	M	28.4	17867	9	1.8	III	v20	<1	NBNC		before operation
HCC	HCC081pre	63	F	4.8	19	ND	ND	I	v20	2<	HCV	no treatment	before operation
HCC	HCC085pre	72	M	5	61	10	2.2	I	v20	2<	NBNC		before operation
HCC	HCC086pre	78	F	24.7	20	44	3.1	I	v20	2<	HCV	no treatment	before operation
HCC	HCC087pre	76	M	5.3	58	13	5.4	II	v20	<1	NBNC		before operation
HCC	HCC088pre	71	M	1231.5	4447	8	3.1	III	v20	<1	HCV	no treatment	before operation
HCC	HCC094pre	59	M	3.5	69	6	1	I	v20	2<	HBV		before operation
HCC	HCC095pre	72	M	3.3	27	ND	ND	I	v20	2<	NBNC		before operation
HCC	HCC100pre	72	M	102.2	1392	8	2.6	I	v20	2<	HCV	no treatment	before operation
HCC	HCC110pre	70	F	12.7	16	ND	ND	I	v20	2<	HCV	no treatment	before operation
HCC	HCC118pre	55	F	486.7	689	ND	ND	III	v20	<1	HCV	SVR	before operation
HCC	HCC120pre	58	M	8.7	280	6	0.9	II	v20	1<<2	HCV	no treatment	before operation
HCC	HCC121pre	65	M	82967	1010	ND	ND	IV	v20	2<	HBV		before operation
HCC	HCC122pre	64	M	4.7	276	6	3.1	I	v20	2<	HCV	no treatment	before operation
HCC	HCC131pre	75	M	154.2	450	90	5.5	II	v20	2<	NBNC		before operation
HCC	HCC133pre	78	F	56.6	29	13	1.5	I	v20	2<	HCV	no treatment	before operation
HCC	HCC136pre	79	F	12	650	24	3.1	I	v20	<1	HCV	no treatment	before operation
HCC	HCC141pre	66	M	5	85	4	1.7	II	v20	2<	HCV	SVR	before operation
HCC	HCC148pre	67	M	26.6	3904	27	4.7	II	v20	1<<2	HCV	no treatment	before operation
HCC	HCC149pre	70	M	2	15	7	1.1	I	v20	2<	HBV		before operation
HCC	HCC150pre	66	M	8.4	32	19	10.4	I	v20	2<	HBV		before operation

HCC	HCC151pre	61	M	4.1	203	82	4.4	III	v20	1<<2	NBNC		before operation
HCC	HCC155pre	76	F	154.6	25	ND	ND	I	v20	<1	ALD		before operation
HCC	HCC159pre	70	M	2.3	822	4	3	I	v20	2<	ALD		before operation
HCC	HCC162pre	56	M	143.1	339	21	21	III	v20	<1	HCV	no treatment	before operation
HCC	HCC163pre	72	F	6.2	25	ND	ND	I	v20	2<	HBV		before operation
HCC	HCC164pre	78	M	543.2	18	11	3.8	III	v20	<1	HCV	no treatment	before operation
HCC	HCC165pre	32	F	105968	2251	15	3	II	v20	<1	HBV		before operation
HCC	HCC167pre	81	M	3.1	19	17	3.8	I	v20	2<	HCV	no treatment	before operation
HCC	HCC168pre	72	M	2.9	14	7	1.4	I	v20	2<	NBNC		before operation
HCC	HCC169pre	75	M	7	18	9	2.2	I	v20	2<	HCV	no treatment	before operation
HCC	HCC170pre	74	M	4.6	24	2	7.2	II	v20	<1	HCV	SVR	before operation
HCC	HCC174pre	78	M	4.1	259	ND	ND	III	v20	2<	HCV	no treatment	before operation
HCC	HCC177pre	60	M	3.6	18	ND	ND	I	v20	2<	HBV		before operation
HCC	HCC180pre	45	M	578.5	39	ND	ND	I	v20	2<	HBV		before operation
HCC	HCC182pre	77	M	4.2	54	7	3.9	I	v20	2<	HBV		before operation
HCC	HCC188pre	77	F	3.5	19	ND	ND	II	v20	2<	HCV	no treatment	before operation
HCC	HCC192pre	64	M	91	77	ND	ND	II	v20	<1	ALD		before operation
HCC	HCC193pre	77	F	9.2	566	5	9.3	I	v20	<1	HCV	no treatment	before operation
HCC	HCC197pre	77	M	8	26	ND	ND	II	v20	2<	HCV	no treatment	before operation
HCC	HCC198pre	64	M	2.3	32	ND	ND	I	v20	1<<2	HBV		before operation
HCC	HCC199pre	57	M	1741.6	4614	3	13	I	v20	<1	HBV		before operation
HCC	HCC210pre	79	F	8.9	19	ND	ND	I	v20	2<	HCV	no treatment	before operation
HCC	HCC211pre	80	M	107.6	80	10	4.4	I	v20	<1	HCV	no treatment	before operation
HCC	HCC217pre	72	F	9.2	21	44	1.5	II	v20	1<<2	HCV	no treatment	before operation
HCC	HCC218pre	47	M	26791	3400	8	0.8	IV	v20	<1	NBNC		before operation
HCC	HCC221pre	72	F	9.2	21	44	1.5	II	v20	2<	HCV	no treatment	before operation
HCC	HCC223pre	68	M	5.9	171	5	5.9	II	v20	2<	ALD		before operation
HCC	HCC226pre	71	M	162.4	531292	2	4	III	v20	2<	HCV	no treatment	before operation
HCC	HCC230pre	65	M	2	25	ND	ND	I	v20	2<	HCV	SVR	before operation
HCC	HCC024pre-2	71	F	10.8	18	ND	ND	I	v20	2<	HCV	no treatment	before operation at 2nd recurrence
HCC	HCC034pre-2	60	M	103.8	1220	ND	ND	III	v20	1<<2	NBNC		before operation at 2nd recurrence
HCC	HCC060pre-2	78	F	1078.4	847	ND	ND	II	v20	<1	HCV	SVR	before operation at 2nd recurrence
HCC	HCC006post1	69	F	862.9	909	ND	ND	I	v20	<1	NBNC		after operation within 14 days
HCC	HCC018post1	62	M	989.5	93762	ND	ND	ND	v20	2<	HCV	no treatment	after operation within 14 days
HCC	HCC019post1	65	M	8.1	40	ND	4.7	I	v20	2<	HBV		after operation within 14 days
HCC	HCC024post1	76	M	2.3	12	6	1.3	I	v20	2<	HCV	SVR	after operation within 14 days
HCC	HCC026post1	70	F	10.8	18	ND	ND	I	v20	2<	HCV	no treatment	after operation within 14 days
HCC	HCC028post1	72	M	3.6	112	ND	ND	I	v20	2<	HCV	no treatment	after operation within 14 days
HCC	HCC032post1	74	F	30.2	10	21	3.2	I	v20	2<	HCV	no treatment	after operation within 14 days
HCC	HCC034post1	60	M	103.8	1220	ND	ND	III	v20	2<	NBNC		after operation within 14 days
HCC	HCC037post1	81	M	4.5	191	4	7.2	III	v20	<1	HCV	no treatment	after operation within 14 days
HCC	HCC038post1	79	M	52.2	1000	5	1	II	v20	2<	HCV	no treatment	after operation within 14 days
HCC	HCC040post1	66	M	1113.3	3148	18	1.5	IV	v20	<1	HBV		after operation within 14 days
HCC	HCC041post1	76	M	4	333	9	ND	II	v20	1<<2	ALD		after operation within 14 days
HCC	HCC043post1	60	F	1.8	16	ND	ND	I	v20	2<	HCV	no treatment	after operation within 14 days
HCC	HCC044post1	45	F	1264.4	4831	ND	ND	III	v20	2<	HBV		after operation within 14 days
HCC	HCC046post1	64	M	3.7	21	9	2.8	II	v20	2<	HBV		after operation within 14 days
HCC	HCC050post1	63	F	3.5	27	10	0.6	II	v20	2<	NBNC		after operation within 14 days
HCC	HCC051post1	63	M	3.1	12	4	2	II	v20	2<	NBNC		after operation within 14 days
HCC	HCC052post1	75	M	4	5190	16	1.6	III	v20	<1	HCV	no treatment	after operation within 14 days
HCC	HCC053post1	51	M	3.9	19	12	1.4	II	v20	<1	HBV		after operation within 14 days
HCC	HCC054post1	69	M	52.7	58	7	1.4	I	v20	2<	HBV		after operation within 14 days
HCC	HCC058post1	70	M	3	34	ND	ND	I	v20	1<<2	HBV		after operation within 14 days
HCC	HCC060post1	77	F	1078.4	847	ND	ND	II	v20	2<	HCV	SVR	after operation within 14 days

HCC	HCC061post1	77	F	56.6	29	13	1.5	I	v20	<1	HBV		after operation within 14 days
HCC	HCC066post1	63	M	217.2	403	ND	ND	III	v20	<1	HCV	no treatment	after operation within 14 days
HCC	HCC070post1	67	M	20	59	6	1.4	I	v20	2<	HBV		after operation within 14 days
HCC	HCC071post1	49	M	5.9	21	12	5.4	I	v20	2<	HCV	no treatment	after operation within 14 days
HCC	HCC072post1	65	M	10.1	28	ND	ND	I	v20	2<	HCV	SVR	after operation within 14 days
HCC	HCC074post1	67	M	2.5	37	61	3.9	II	v20	2<	HCV	SVR	after operation within 14 days
HCC	HCC075post1	77	F	12	14	ND	ND	I	v20	1<<2	HCV	no treatment	after operation within 14 days
HCC	HCC076post1	76	M	28.4	17867	9	1.8	III	v20	<1	NBNC		after operation within 14 days
HCC	HCC081post1	63	F	4.8	19	ND	ND	I	v20	2<	HCV	no treatment	after operation within 14 days
HCC	HCC085post1	72	M	5	61	10	2.2	I	v20	2<	NBNC		after operation within 14 days
HCC	HCC086post1	78	F	24.7	20	44	3.1	I	v20	2<	HCV	no treatment	after operation within 14 days
HCC	HCC087post1	76	M	5.3	58	13	5.4	II	v20	<1	NBNC		after operation within 14 days
HCC	HCC088post1	71	M	1231.5	4447	8	3.1	III	v20	<1	HCV	no treatment	after operation within 14 days
HCC	HCC094post1	59	M	3.5	69	6	1	I	v20	2<	HBV		after operation within 14 days
HCC	HCC095post1	72	M	3.3	27	ND	ND	I	v20	2<	NBNC		after operation within 14 days
HCC	HCC100post1	72	M	102.2	1392	8	2.6	I	v20	2<	HCV	no treatment	after operation within 14 days
HCC	HCC110post1	70	F	12.7	16	ND	ND	I	v20	2<	HCV	no treatment	after operation within 14 days
HCC	HCC118post1	55	F	486.7	689	ND	ND	III	v20	<1	HCV	SVR	after operation within 14 days
HCC	HCC120post1	58	M	8.7	280	6	0.9	II	v20	1<<2	HCV	no treatment	after operation within 14 days
HCC	HCC121post1	65	M	82967	1010	ND	ND	IV	v20	2<	HBV		after operation within 14 days
HCC	HCC131post1	75	M	154.2	450	90	5.5	II	v20	2<	NBNC		after operation within 14 days
HCC	HCC133post1	78	F	56.6	29	13	1.5	I	v20	2<	HCV	no treatment	after operation within 14 days
HCC	HCC136post1	79	F	12	650	24	3.1	I	v20	<1	HCV	no treatment	after operation within 14 days
HCC	HCC141post1	67	M	5	85	4	1.7	II	v20	2<	HCV	SVR	after operation within 14 days
HCC	HCC148post1	67	M	26.6	3904	27	4.7	II	v20	<1	HCV	no treatment	after operation within 14 days
HCC	HCC149post1	70	M	2	15	7	1.1	I	v20	2<	HBV		after operation within 14 days
HCC	HCC150post1	66	M	8.4	32	19	10.4	I	v20	2<	HBV		after operation within 14 days
HCC	HCC151post1	61	M	4.1	203	82	4.4	III	v20	1<<2	NBNC		after operation within 14 days
HCC	HCC155post1	76	F	154.6	25	ND	ND	I	v20	<1	ALD		after operation within 14 days
HCC	HCC159post1	70	M	2.3	822	4	3	I	v20	2<	ALD		after operation within 14 days
HCC	HCC162post1	56	M	143.1	339	21	21	III	v20	<1	HCV	no treatment	after operation within 14 days
HCC	HCC163post1	72	F	6.2	25	ND	ND	I	v20	2<	HBV		after operation within 14 days
HCC	HCC164post1	78	M	543.2	18	11	3.8	III	v20	<1	HCV	no treatment	after operation within 14 days
HCC	HCC167post1	81	M	3.1	19	17	3.8	I	v20	2<	HCV	no treatment	after operation within 14 days
HCC	HCC168post1	72	M	2.9	14	7	1.4	I	v20	2<	NBNC		after operation within 14 days
HCC	HCC169post1	75	M	7	18	9	2.2	I	v20	2<	HCV	no treatment	after operation within 14 days
HCC	HCC170post1	76	M	4.6	24	2	7.2	II	v20	<1	HCV	SVR	after operation within 14 days
HCC	HCC174post1	78	M	4.1	259	ND	ND	III	v20	2<	HCV	no treatment	after operation within 14 days
HCC	HCC177post1	60	M	3.6	18	ND	ND	I	v20	2<	HBV		after operation within 14 days
HCC	HCC180post1	45	M	578.5	39	ND	ND	I	v20	2<	HBV		after operation within 14 days
HCC	HCC182post1	77	M	4.2	54	7	3.9	I	v20	2<	HBV		after operation within 14 days
HCC	HCC188post1	77	F	3.5	19	ND	ND	II	v20	2<	HCV	no treatment	after operation within 14 days
HCC	HCC197post1	77	M	8	26	ND	ND	II	v20	2<	HCV	no treatment	after operation within 14 days
HCC	HCC198post1	64	M	2.3	32	ND	ND	I	v20	1<<2	HBV		after operation within 14 days
HCC	HCC199post1	57	M	1741.6	4614	3	13	I	v20	<1	HBV		after operation within 14 days
HCC	HCC210post1	79	F	8.9	19	ND	ND	I	v20	2<	HCV	no treatment	after operation within 14 days
HCC	HCC211post1	80	M	107.6	80	10	4.4	I	v20	<1	HCV	no treatment	after operation within 14 days
HCC	HCC217post1	72	F	9.2	21	44	1.5	II	v20	1<<2	HCV	no treatment	after operation within 14 days
HCC	HCC218post1	47	M	26791	3400	8	0.8	IV	v20	<1	NBNC		after operation within 14 days
HCC	HCC223post1	68	M	5.9	171	5	5.9	II	v20	2<	ALD		after operation within 14 days
HCC	HCC226post1	71	M	162.4	531292	2	4	III	v20	2<	HCV	no treatment	after operation within 14 days
HCC	HCC230post1	65	M	2	25	ND	ND	I	v20	2<	HCV	SVR	after operation within 14 days
HCC	HCC002post2	81	F	42.9	609	5	2.8	I	v20	<1	HCV	no treatment	after operation beyand 14 days
HCC	HCC003post2	77	F	18.6	41	10	1.4	III	v20	2<	HBV		after operation beyand 14 days

HCC	HCC006post2	69	F	862.9	909	ND	ND	I	v20	<1	NBNC		after operation beyand 14 days
HCC	HCC018post2	62	M	989.5	93762	ND	ND	ND	v20	2<	HCV	no treatment	after operation beyand 14 days
HCC	HCC019post2	65	M	8.1	40	ND	4.7	I	v20	2<	HBV		after operation beyand 14 days
HCC	HCC024post2	76	M	2.3	12	6	1.3	I	v20	2<	HCV	SVR	after operation beyand 14 days
HCC	HCC028post2	72	M	3.6	112	ND	ND	I	v20	2<	HCV	no treatment	after operation beyand 14 days
HCC	HCC032post2	73	F	30.2	10	21	3.2	I	v20	2<	HCV	no treatment	after operation beyand 14 days
HCC	HCC034post2	60	M	103.8	1220	ND	ND	III	v20	2<	NBNC		after operation beyand 14 days
HCC	HCC037post2	80	M	4.5	191	4	7.2	III	v20	<1	HCV	no treatment	after operation beyand 14 days
HCC	HCC038post2	79	M	52.2	1000	5	1	II	v20	2<	HCV	no treatment	after operation beyand 14 days
HCC	HCC040post2	65	M	1113.3	3148	18	1.5	IV	v20	<1	HBV		after operation beyand 14 days
HCC	HCC041post2	76	M	4	333	9	ND	II	v20	1<<2	ALD		after operation beyand 14 days
HCC	HCC043post2	60	F	1.8	16	ND	ND	I	v20	2<	HCV	no treatment	after operation beyand 14 days
HCC	HCC044post2	45	F	1264.4	4831	ND	ND	III	v20	2<	HBV		after operation beyand 14 days
HCC	HCC046post2	63	M	3.7	21	9	2.8	II	v20	2<	HBV		after operation beyand 14 days
HCC	HCC049post2	49	M	7.2	46	ND	ND	I	v20	<1	ALD		after operation beyand 14 days
HCC	HCC051post2	77	M	3.1	12	4	2	II	v20	2<	NBNC		after operation beyand 14 days
HCC	HCC052post2	75	M	4	5190	16	1.6	III	v20	<1	HCV	no treatment	after operation beyand 14 days
HCC	HCC053post2	51	M	3.9	19	12	1.4	II	v20	<1	HBV		after operation beyand 14 days
HCC	HCC054post2	69	M	52.7	58	7	1.4	I	v20	2<	HBV		after operation beyand 14 days
HCC	HCC058post2	71	M	3	34	ND	ND	I	v20	1<<2	HBV		after operation beyand 14 days
HCC	HCC066post2	63	M	217.2	403	ND	ND	III	v20	<1	HCV	no treatment	after operation beyand 14 days
HCC	HCC072post2	65	M	10.1	28	ND	ND	I	v20	2<	HCV	SVR	after operation beyand 14 days
HCC	HCC074post2	67	M	2.5	37	61	3.9	II	v20	2<	HCV	SVR	after operation beyand 14 days
HCC	HCC075post2	77	F	12	14	ND	ND	I	v20	1<<2	HCV	no treatment	after operation beyand 14 days
HCC	HCC076post2	76	M	28.4	17867	9	1.8	III	v20	<1	NBNC		after operation beyand 14 days
HCC	HCC081post2	63	F	4.8	19	ND	ND	I	v20	2<	HCV	no treatment	after operation beyand 14 days
HCC	HCC085post2	72	M	5	61	10	2.2	I	v20	2<	NBNC		after operation beyand 14 days
HCC	HCC086post2	78	F	24.7	20	44	3.1	I	v20	2<	HCV	no treatment	after operation beyand 14 days
HCC	HCC088post2	71	M	1231.5	4447	8	3.1	III	v20	<1	HCV	no treatment	after operation beyand 14 days
HCC	HCC095post2	72	M	3.3	27	ND	ND	I	v20	2<	NBNC		after operation beyand 14 days
HCC	HCC110post2	70	F	12.7	16	ND	ND	I	v20	2<	HCV	no treatment	after operation beyand 14 days
HCC	HCC120post2	59	M	8.7	280	6	0.9	II	v20	1<<2	HCV	no treatment	after operation beyand 14 days
HCC	HCC121post2	65	M	82967	1010	ND	ND	IV	v20	2<	HBV		after operation beyand 14 days
HCC	HCC122post2	64	M	4.7	276	6	3.1	I	v20	2<	HCV	SVR	after operation beyand 14 days
HCC	HCC133post2	78	F	56.6	29	13	1.5	I	v20	2<	HCV	no treatment	after operation beyand 14 days
HCC	HCC151post2	61	M	4.1	203	82	4.4	III	v20	1<<2	NBNC		after operation beyand 14 days
HCC	HCC163post2	72	F	6.2	25	ND	ND	I	v20	2<	HBV		after operation beyand 14 days
HCC	HCC164post2	78	M	543.2	18	11	3.8	III	v20	<1	HCV	no treatment	after operation beyand 14 days
HCC	HCC165post2	32	F	105968	2251	15	3	II	v20	<1	HBV		after operation beyand 14 days
HCC	HCC192post2	64	M	91	77	ND	ND	II	v20	<1	ALD		after operation beyand 14 days
HCC	HCC193post2	77	F	9.2	566	5	9.3	I	v20	<1	HCV	no treatment	after operation beyand 14 days
HCC	HCC210post2	79	F	8.9	19	ND	ND	I	v20	2<	HCV	no treatment	after operation beyand 14 days
HCC	HCC218post2	48	M	26791	3400	8	0.8	IV	v20	<1	NBNC		after operation beyand 14 days
HCC	HCC221post2	72	F	9.2	21	44	1.5	II	v20	2<	HCV	no treatment	after operation beyand 14 days
HCC	HCC226post2	71	M	162.4	531292	2	4	III	v20	2<	HCV	no treatment	after operation beyand 14 days
HCC	HCC013pre	73	F	5.7	38	1130	2.7	IV	v21	2<	HBV		before operation
HCC	HCC258pre	73	F	52.3	40	5	2.3	I	v21	<1	NBNC		before operation
HCC	HCC296pre	36	F	56.6	29	13	1.5	I	v21	ND	NBNC		before operation
HCC	HCC334pre	57	M	355.3	7516	ND	ND	ND	v21	ND	HCV	no treatment	before operation
HCC	HCC349pre	77	M	2	17	12	2.8	I	v21	2<	NBNC		before operation
HCC	HCC355pre	77	M	4.2	27	5	ND	I	v21	1<<2	NBNC		before operation
HCC	HCC380pre	81	F	9.5	20	8	3.5	I	v21	1<<2	HCV	no treatment	before operation
HCC	HCC394pre	66	M	4.3	16262	9	3.4	IV	v21	2<	HBV		before operation
HCC	HCC399pre	74	M	35	1658	ND	86.1	II	v21	ND	HCV	no treatment	before operation
HCC	HCC415pre	81	M	79.9	144	12	1.6	II	v21	<1	HCV	no treatment	before operation

HCC	HCC420pre	68	M	23.4	7078	10	4.8	I	v21	2<	HCV	no treatment	before operation
HCC	HCC423pre	64	M	6	47	11	2.5	I	v21	2<	HCV	no treatment	before operation
HCC	HCC434pre	71	M	8.1	18	ND	ND	II	v21	2<	HCV	no treatment	before operation
HCC	HCC449pre	76	M	775.3	289	22	3.1	I	v21	2<	HBV		before operation
HCC	HCC460pre	77	M	2	26	ND	ND	II	v21	2<	NBNC		before operation
HCC	HCC467pre	48	M	ND	ND	N26	ND	II	v21	ND	ALD		before operation
HCC	HCC472pre	76	F	3.2	11	ND	ND	I	v21	1<<2	HCV	no treatment	before operation
HCC	HCC476pre	39	M	7.6	22	8	1.8	I	v21	2<	NBNC		before operation
HCC	HCC488pre	81	M	15.5	146	ND	ND	I	v21	<1	HCV	no treatment	before operation
HCC	HCC494pre	80	M	4.2	58	ND	ND	II	v21	<1	HCV	no treatment	before operation
HCC	HCC496pre	82	M	8.8	225	ND	ND	II	v21	<1	HCV	no treatment	before operation
HCC	HCC497pre	84	M	8.8	225	ND	ND	II	v21	<1	HCV	no treatment	before operation
HCC	HCC505pre	78	F	6.8	88	22	2.9	I	v21	2<	HBV		before operation
HCC	HCC508pre	73	M	6.2	3656	8	11.2	III	v21	1<<2	ALD		before operation
HCC	HCC509pre	53	M	826.2	2391	8	1.4	II	v21	2<	HBV		before operation
HCC	HCC510pre	69	F	44.3	56	ND	ND	I	v21	ND	HBV		before operation
HCC	HCC515pre	58	F	1440.9	871	ND	ND	III	v21	2<	HBV		before operation
HCC	HCC538pre	70	M	8.4	130	ND	ND	II	v21	2<	HCV	no treatment	before operation
HCC	HCC545pre	78	M	2.4	21	ND	ND	I	v21	2<	HBV		before operation
HCC	HCC553pre	48	F	3	67.5	14	1	I	v21	1<<2	HCV	no treatment	before operation
HCC	HCC568pre	85	M	12.1	100	6	3.7	I	v21	1<<2	HCV	no treatment	before operation
HCC	HCC571pre	79	F	2.6	1907	6	3.4	I	v21	2<	HCV	no treatment	before operation
HCC	HCC575pre	78	M	2.4	14	ND	ND	II	v21	2<	NBNC		before operation
HCC	HCC580pre	76	M	129.5	874	48	3.7	IV	v21	<1	HBV		before operation
HCC	HCC583pre	72	M	4	156	ND	ND	I	v21	2<	NBNC		before operation
HCC	HCC588pre	77	F	8.7	52	12	5.9	I	v21	<1	HBV		before operation
HCC	HCC590pre	78	F	6.4	16	2	2.5	I	v21	2<	HCV	no treatment	before operation
HCC	HCC591pre	77	M	3.6	21	64	4.7	I	v21	2<	HCV	no treatment	before operation
HCC	HCC592pre	74	F	24.6	33	ND	ND	II	v21	2<	HCV	no treatment	before operation
HCC	HCC593pre	79	F	7.5	20	25	2.8	I	v21	2<	HCV	no treatment	before operation
HCC	HCC600pre	80	M	7.5	12	8	2.5	II	v21	1<<2	HCV	no treatment	before operation
HCC	HCC601pre	68	M	418180	7028	2	1.4	III	v21	<1	HCV	no treatment	before operation
HCC	HCC602pre	56	F	666	159	49	3.9	I	v21	1<<2	ALD		before operation
HCC	HCC604pre	65	M	4.8	260	3	3.2	II	v21	2<	HCV	SVR	before operation
HCC	HCC606pre	47	M	2.7	168	12	3.1	III	v21	1<<2	HCV	no treatment	before operation
HCC	HCC607pre	81	F	2.1	22	5	3	I	v21	1<<2	NBNC		before operation
HCC	HCC608pre	84	F	817.5	47	17	5.5	I	v21	2<	HCV	no treatment	before operation
HCC	HCC609pre	69	M	4.4	78	ND	ND	I	v21	1<<2	HCV	no treatment	before operation
HCC	HCC611pre	66	M	7.2	201	12	4.9	I	v21	2<	HCV	SVR	before operation
HCC	HCC612pre	72	M	8.3	25	ND	ND	I	v21	1<<2	HCV	SVR	before operation
HCC	HCC613pre	78	M	4.3	21621	7	8.5	IV	v21	1<<2	HCV	no treatment	before operation
HCC	HCC617pre	65	M	5.7	18	2	ND	I	v21	2<	HCV	no treatment	before operation
HCC	HCC618pre	68	M	12	416	4	2	III	v21	2<	HBV+HCV		before operation
HCC	HCC622pre	82	M	1.7	53	4	3.3	II	v21	1<<2	HCV	no treatment	before operation
HCC	HCC625pre	78	M	19.4	16	785	6.1	I	v21	<1	NBNC		before operation
HCC	HCC628pre	ND	M	7	32	5.4	12	I	v21	1<<2	HCV	SVR	before operation
HCC	HCC631pre	70	F	23874	919	18	1.6	IV	v21	1<<2	NBNC		before operation
HCC	HCC632pre	68	M	220.1	24	34	5.5	I	v21	2<	HCV	no treatment	before operation
HCC	HCC633pre	68	M	10.9	21	20	ND	II	v21	2<	HCV	no treatment	before operation
HCC	HCC637pre	65	M	3.2	201	6	2.7	III	v21	1<<2	HBV		before operation
HCC	HCC638pre	79	M	11.6	14	2	3.5	I	v21	2<	HCV	no treatment	before operation
HCC	HCC639pre	75	M	3.5	1032	21	8.7	I	v21	2<	NBNC		before operation
HCC	HCC641pre	72	M	5.6	63	8	ND	II	v21	<1	NBNC		before operation
HCC	HCC642pre	43	M	4.4	16	4	3	ND	v21	2<	HCV	no treatment	before operation
HCC	HCC643pre	72	F	82.9	316	ND	ND	IV	v21	<1	HCV	no treatment	before operation

HCC	HCC644pre	72	F	82.9	316	ND	ND	IV	v21	<1	HCV	no treatment	before operation
HCC	HCC645pre	76	M	2.6	24	23	1.6	II	v21	2<	NBNC		before operation
HCC	HCC646pre	53	M	11.3	16	5	4.7	I	v21	2<	HCV	no treatment	before operation
HCC	HCC647pre	73	F	17.6	44	ND	ND	II	v21	<1	HCV	no treatment	before operation
HCC	HCC648pre	70	M	5.5	25	22	5.2	I	v21	<1	HCV	no treatment	before operation
HCC	HCC649pre	76	M	5.5	16	2	3.2	IV	v21	<1	HCV	no treatment	before operation
HCC	HCC650pre	65	M	2.7	74	3	2.7	II	v21	2<	HBV		before operation
HCC	HCC652pre	75	F	8.2	49	ND	ND	I	v21	1<<2	HCV	no treatment	before operation
HCC	HCC653pre	50	M	4.3	28	7	0.5	I	v21	2<	NBNC		before operation
HCC	HCC654pre	79	M	747.5	39589	2	ND	II	v21	2<	HCV	no treatment	before operation
HCC	HCC655pre	81	M	7.5	12	8	2.5	II	v21	1<<2	HCV	no treatment	before operation
HCC+ICC	HCC657pre	81	M	2	36	6	3.5	II	v21	<1	HCV	SVR	before operation
HCC	HCC659pre	74	M	406	547	7	1.7	I	v21	2<	HCV	no treatment	before operation
HCC	HCC660pre	71	M	34.7	28	2	3.6	I	v21	ND	HCV	no treatment	before operation
HCC	HCC661pre	87	M	117.3	20	6	3.4	III	v21	1<<2	HBV		before operation
HCC	HCC662pre	49	M	5.4	429	ND	ND	II	v21	2<	NBNC		before operation
HCC	HCC664pre	79	M	13.2	28	23	5.3	I	v21	2<	HCV	no treatment	before operation
HCC	HCC665pre	78	M	4.3	19	3	1.5	I	v21	2<	HCV	no treatment	before operation
HCC	HCC670pre	74	F	3.3	216	ND	ND	II	v21	<1	HCV	no treatment	before operation
HCC	HCC671pre	37	F	1078.4	847	ND	ND	II	v21	ND	HCV	SVR	before operation
HCC	HCC673pre	78	M	8.5	190	5	2.8	II	v21	<1	NBNC		before operation
HCC	HCC674pre	74	M	927.3	8321	ND	ND	III	v21	1<<2	HCV	SVR	before operation
HCC	HCC676pre	50	M	4	5220	47	1.5	I	v21	2<	HBV		before operation
HCC	HCC678pre	43	F	1216.9	1070	ND	ND	IV	v21	<1	HCV	no treatment	before operation
HCC	HCC679pre	72	M	3.9	91	3.1	4.5	II	v21	1<<2	NBNC		before operation
HCC	HCC680pre	67	F	21.9	9	17	4	I	v21	2<	HCV	no treatment	before operation
HCC	HCC682pre	69	M	2	21	3	2.2	II	v21	2<	HBV		before operation
HCC	HCC683pre	67	M	5.6	166	17	3.6	II	v21	1<<2	NBNC		before operation
HCC	HCC684pre	75	M	3	19	6	1.8	I	v21	<1	HCV	no treatment	before operation
HCC	HCC685pre	48	F	125	17	4	0.8	II	v21	2<	HCV	SVR	before operation
HCC	HCC687pre	74	M	133.1	40	2	4.1	I	v21	<1	HCV	SVR	before operation
HCC	HCC688pre	60	F	130.5	1040	4	0.5	I	v21	2<	NBNC		before operation
HCC	HCC689pre	59	M	11.9	248	6	2.5	III	v21	1<<2	HCV	no treatment	before operation
HCC	HCC690pre	62	F	3	61	3	1.2	I	v21	2<	HBV		before operation
HCC	HCC691pre	75	F	6.2	42	ND	ND	I	v21	<1	HCV	no treatment	before operation
HCC	HCC692pre	72	M	2	49	ND	ND	II	v21	2<	HBV		before operation
HCC	HCC697pre	58	M	3.1	19	ND	ND	I	v21	2<	HCV	SVR	before operation
HCC	HCC699pre	75	F	2.6	16	38	2.7	I	v21	1<<2	HCV	no treatment	before operation
HCC	HCC700pre	69	M	3.1	33	ND	ND	II	v21	2<	HCV	SVR	before operation
HCC	HCC702pre	71	M	2.4	22	3	3.8	II	v21	2<	NBNC		before operation
HCC	HCC703pre	76	F	33.5	1162	ND	ND	II	v21	2<	HCV	no treatment	before operation
HCC	HCC704pre	69	M	3	698	ND	ND	IV	v21	<1	HCV	SVR	before operation
HCC	HCC705pre	58	F	4.1	61	2	ND	III	v21	<1	NBNC		before operation
HCC	HCC706pre	62	M	12.8	16021	4	4.3	III	v21	1<<2	HBV+HCV		before operation
HCC	HCC707pre	50	F	73.4	1993	6	5.4	III	v21	1<<2	HCV	no treatment	before operation
HCC	HCC709pre	72	F	26.6	43934	10	3.6	I	v21	<1	HCV	SVR	before operation
HCC	HCC711pre	81	M	3.8	216	8	1.8	II	v21	1<<2	ALD		before operation
HCC	HCC713pre	74	F	1363	15817	21	12.3	III	v21	2<	HCV	no treatment	before operation
HCC	HCC714pre	67	M	453.9	58703	12	2.5	III	v21	<1	NBNC		before operation
HCC	HCC715pre	75	M	5.6	48	4	2.3	III	v21	1<<2	NBNC		before operation
HCC	HCC716pre	74	F	11.5	31	13	4.6	I	v21	2<	HCV	no treatment	before operation
HCC	HCC717pre	69	F	5	22	21	3.9	I	v21	2<	HCV	SVR	before operation
HCC	HCC718pre	82	M	3.1	5	5	2.1	I	v21	2<	HCV	no treatment	before operation
HCC	HCC720pre	66	M	4.4	44	6	1.5	I	v21	<1	HBV		before operation
HCC	HCC721pre	76	M	8.2	16480	7	ND	II	v21	ND	HBV		before operation

HCC	HCC722pre	78	M	86.7	20450	8	1.7	II	v21	1<<2	NBNC		before operation
HCC	HCC726pre	67	M	3.4	61	7	1.7	I	v21	<1	NBNC		before operation
HCC	HCC730pre	67	M	4	20326	ND	ND	IV	v21	1<<2	ALD		before operation
HCC	HCC732pre	63	M	6.7	26	5	2.8	I	v21	2<	HCV	SVR	before operation
HCC	HCC733pre	73	M	2.6	134	16	3.2	I	v21	1<<2	HBV		before operation
HCC	HCC735pre	74	M	7.7	392	2	ND	I	v21	1<<2	HBV+HCV		before operation
HCC	HCC736pre	80	F	119.9	401	21	2.7	II	v21	1<<2	HCV	no treatment	before operation
HCC	HCC737pre	64	M	3.4	292	2	3	I	v21	2<	NBNC		before operation
HCC	HCC738pre	76	M	4	33	14	3.3	II	v21	1<<2	HBV		before operation
HCC	HCC739pre	84	F	24.4	22	2	1.4	I	v21	2<	NBNC		before operation
HCC	HCC740pre	75	M	4.3	591	12	5	II	v21	2<	HBV		before operation
HCC	HCC742pre	70	M	5	129	2	2.8	II	v21	2<	HCV	no treatment	before operation
HCC	HCC748pre	79	M	3.5	49	4	3.2	I	v21	2<	NBNC		before operation
HCC	HCC750pre	60	M	22.6	19	ND	ND	I	v21	<1	HBV		before operation
HCC	HCC751pre	83	M	3	3089	2	1.1	I	v21	2<	HCV	SVR	before operation
HCC	HCC752pre	49	M	166.3	101	11	2.8	II	v21	<1	HBV		before operation
HCC	HCC753pre	59	M	8.6	354	17	4	II	v21	<1	NBNC		before operation
HCC	HCC761pre	75	M	4641	61352	5	0.9	II	v21	1<<2	NBNC		before operation
HCC	HCC763pre	49	M	72325	5503	ND	ND	III	v21	2<	HBV		before operation
HCC	HCC764pre	65	M	459.9	8326	15	4	II	v21	<1	HCV	SVR	before operation
HCC	HCC766pre	60	M	74.9	133	12	2.3	I	v21	2<	HBV		before operation
HCC	HCC769pre	60	M	29.7	76	ND	ND	I	v21	1<<2	HCV	SVR	before operation
HCC	HCC774pre	55	M	7	15	19	2.4	I	v21	2<	HBV		before operation
HCC	HCC775pre	49	M	166.3	101	11	2.8	II	v21	2<	HBV		before operation
HCC	HCC778pre	66	F	2	144	2	1.9	I	v21	2<	HBV		before operation
HCC	HCC780pre	75	M	3.6	20	9	1.3	II	v21	2<	HCV	SVR	before operation
HCC	HCC785pre	73	M	925.1	81597	2	1.7	III	v21	2<	NBNC		before operation
HCC	HCC786pre	83	M	1035.5	88	< 2	4.2	III	v21	<1	HCV	no treatment	before operation
HCC	HCC787pre	47	M	829.1	105	3	1.4	IV	v21	ND	NBNC		before operation
HCC	HCC788pre	71	M	24.2	40078	<2	1.4	II	v21	1<<2	HCV	no treatment	before operation
HCC	HCC789pre	75	M	4.4	23	9	3	I	v21	2<	HBV		before operation
HCC	HCC790pre	71	M	9.5	12	ND	ND	I	v21	2<	HCV	no treatment	before operation
HCC	HCC791pre	71	M	2.5	5222	2	3.3	II	v21	<1	NBNC		before operation
HCC	HCC792pre	66	F	9.5	19	ND	ND	II	v21	1<<2	NBNC		before operation
HCC	HCC794pre	78	M	51.5	26	ND	6.5	II	v21	1<<2	HCV	no treatment	before operation
HCC	HCC795pre	68	F	215.8	15	2	1.9	II	v21	<1	HCV	no treatment	before operation
HCC	HCC798pre	72	F	2.7	1331	2	2.8	III	v21	2<	HBV		before operation
HCC	HCC799pre	74	M	16.6	66	ND	ND	I	v21	1<<2	HCV	SVR	before operation
HCC	HCC800pre	78	F	33.1	12584	4	2.1	II	v21	<1	NBNC		before operation
HCC	HCC801pre	68	M	3.1	19879	ND	ND	III	v21	2<	HBV		before operation
HCC	HCC802pre	64	M	69.5	168	2	1.6	II	v21	2<	HCV	no treatment	before operation
HCC	HCC803pre	68	M	2	746	ND	ND	II	v21	1<<2	HBV		before operation
HCC	HCC804pre	74	M	60.6	33	ND	ND	I	v21	2<	HCV	SVR	before operation
HCC	HCC806pre	59	M	10.1	457	5	3.1	III	v21	<1	HCV	no treatment	before operation
HCC	HCC807pre	77	M	3.3	49	11	1.7	I	v21	2<	NBNC		before operation
HCC	HCC809pre	82	M	2.4	41	ND	ND	I	v21	2<	HCV	SVR	before operation
HCC	HCC810pre	81	M	23.8	39	56	5.5	II	v21	2<	HCV	no treatment	before operation
HCC	HCC811pre	ND	M	2.4	310	9	2.3	II	v21	<1	HBV		before operation
HCC	HCC812pre	68	M	1.7	87	ND	ND	II	v21	2<	NBNC		before operation
HCC	HCC813pre	72	M	63.1	3219	9	7.9	II	v21	2<	NBNC		before operation
HCC	HCC814pre	81	M	5.5	56	4	2.2	I	v21	2<	NBNC		before operation
HCC	HCC815pre	77	M	3.7	16	9	2.1	II	v21	2<	HCV	no treatment	before operation
HCC	HCC816pre	65	M	3.5	ND	10	2.8	II	v21	2<	HBV		before operation
HCC	HCC817pre	63	M	9.1	74	ND	ND	ND	v21	2<	NBNC		before operation
HCC	HCC818pre	67	F	52.6	26	7	3.4	II	v21	2<	NBNC		before operation

HCC	HCC026pre-2	55	M	4.8	47	10	18.1	ND	v21	2<	HBV	before operation at 2nd recurrence
HCC	HCC051pre-2	55	M	5	583	24	4.5	III	v21	<1	HBV	before operation at 2nd recurrence
ICC	ICC004pre	76	M	ND	ND	836	9.7	IV	v21	2<	ND	before operation
ICC	ICC077pre	66	F	ND	ND	147	ND	III	v21	2<	ND	before operation
ICC	ICC169pre	39	M	ND	ND	82	1.7	IV	v21	2<	ND	before operation
GB cancer	ICC172pre	53	M	3.2	16	3	2.4	IV	v21	ND	ND	before operation
ICC	ICC179pre	31	M	ND	ND	501	2.1	III	v21	2<	ND	before operation
ICC	ICC213pre	31	M	ND	ND	501	2.1	III	v21	2<	ND	before operation
ICC	ICC229pre	ND	ND	ND	ND	ND	ND	ND	v21	ND	ND	before operation
ICC	ICC244pre	35	M	6.7	24	38	3.5	IV	v21	2<	ND	before operation
ICC	ICC267pre	51	F	<2	ND	43566	12.8	IV	v21	ND	ND	before operation
ICC	ICC278pre	63	F	ND	ND	28	3.1	I	v21	2<	ND	before operation
ICC	ICC279pre	77	F	ND	ND	5	2.2	II	v21	2<	ND	before operation
ICC	ICC283pre	35	M	6.7	24	38	3.5	IV	v21	2<	ND	before operation
ICC	ICC285pre	73	F	4.4	11	8	3.5	I	v21	2<	HCV	before operation
ICC	ICC298pre	78	F	ND	ND	14	2.3	II	v21	2<	ND	before operation
ICC	ICC314pre	70	M	ND	ND	45	3.1	IV	v21	ND	ND	before operation
ICC	ICC336pre	68	F	5.3	22	153	3.1	ND	v21	ND	ND	before operation
ICC	ICC404pre	68	M	2.9	12	5.2	0.9	III	v21	2<	ND	before operation
ICC	ICC407pre	80	F	3	53	89	2.1	IV	v21	1<<2	ND	before operation
ICC	ICC409pre	66	M	4.8	15	58	2	IV	v21	2<	ND	before operation
GB cancer	ICC424pre	60	F	3.8	67	51	3.1	IV	v21	ND	ND	before operation
ICC	ICC443pre	66	F	ND	ND	93	3.2	IV	v21	ND	ND	before operation
ICC	ICC534pre	69	M	3.9	12	34	1	II	v21	2<	ND	before operation
ICC	ICC536pre	73	F	2	17	1315	1.4	IV	v21	<1	ND	before operation
ICC	ICC551pre	66	F	ND	ND	15	2	IV	v21	<1	ND	before operation
ICC	ICC594pre	72	M	12	80	11	3.2	III	v21	<1	ALD	before operation
ICC	ICC597pre	58	F	3.2	25	2034	4.1	III	v21	2<	ND	before operation
ICC	ICC614pre	76	F	ND	ND	61	2.2	III	v21	1<<2	ND	before operation
ICC	ICC616pre	68	M	3.1	62	79	2.6	IV	v21	ND	ND	before operation
ICC	ICC619pre	69	M	ND	ND	112	2.5	IV	v21	2<	ND	before operation
ICC	ICC666pre	65	M	31.3	25	5	3.7	III	v21	<1	ND	before operation
ICC	ICC694pre	69	M	39.2	27	9	2.4	II	v21	1<<2	HCV	before operation
ICC	ICC708pre	59	M	16.2	12	3600	8.2	III	v21	2<	ND	before operation
ICC	ICC712pre	75	M	ND	ND	47	4	III	v21	2<	ND	before operation
ICC	ICC762pre	79	M	3.7	13	11	0.6	II	v21	2<	ND	before operation
ICC	ICC768pre	72	M	3.3	40	232	5.3	III	v21	2<	ND	before operation
ICC	ICC777pre	48	F	2	24	15	1.5	IV	v21	2<	ND	before operation
ICC	ICC779pre	68	M	4.2	16	ND	ND	I	v21	2<	ND	before operation
ICC	ICC783pre	68	M	5.4	35	7	1.4	III	v21	2<	ND	before operation
ICC	ICC796pre	78	M	ND	ND	135	23.3	IV	v21	2<	HBV	before operation
ICC	ICC797pre	81	M	3.3	ND	53	2.8	III	v21	1<<2	ND	before operation
ICC	ICC805pre	79	M	4.6	140	543	18.1	IV	v21	ND	ND	before operation
ICC	ICC808pre	56	F	26.5	27	2	4	III	v21	2<	HBV	before operation
ICC	ICC824pre	47	M	13.1	29	1140	3.6	IV	v21	2<	ND	before operation

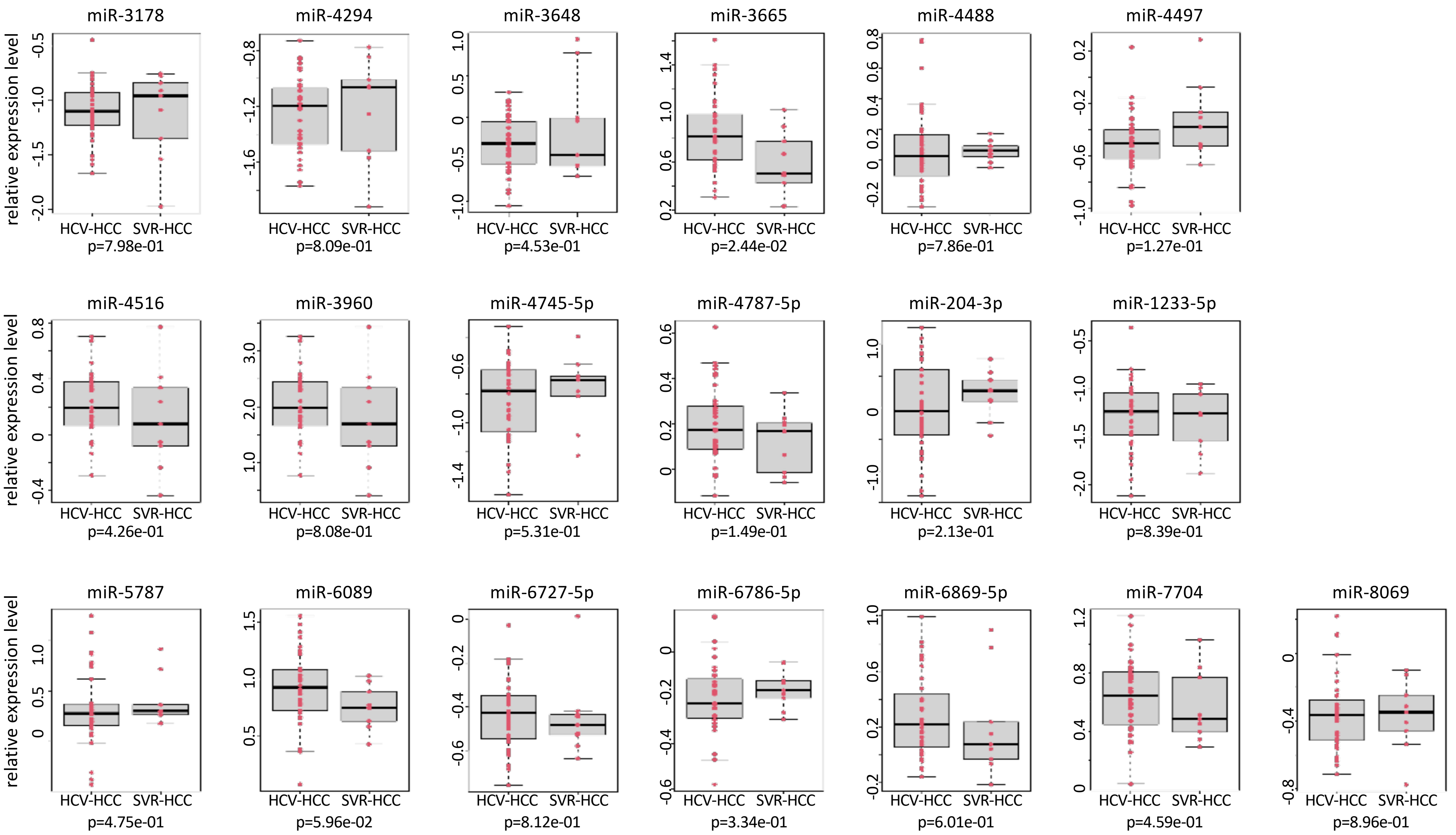

Supplementary figure 1

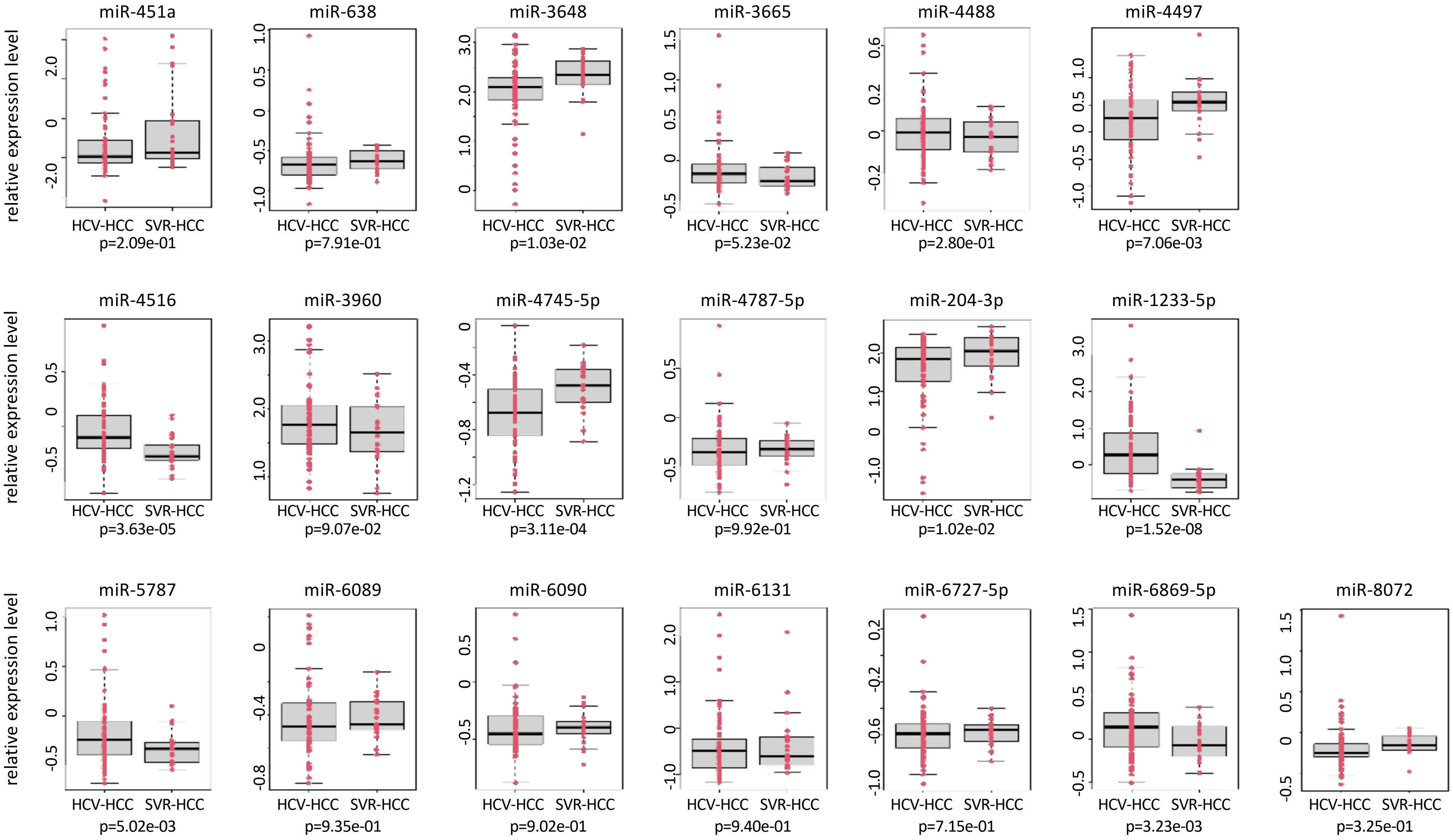

Supplementary figure 2
